# The uric acid-to-HDL ratio as a predictive biomarker for depression risk in adult women

**DOI:** 10.3389/fpsyt.2025.1596708

**Published:** 2025-08-07

**Authors:** Yang Wu, Zhe Wang

**Affiliations:** ^1^ Department of Pharmacy, Heilongjiang Nursing College, Harbin, China; ^2^ The Third Affiliated Hospital of Heilongjiang University of Chinese Medicine, Harbin, China

**Keywords:** uric acid-to-HDL ratio, depression risk, metabolic dysregulation, oxidative stress, women’s mental health

## Abstract

**Background:**

Depression disproportionately affects women, yet biomarkers for early risk stratification remain limited. This study examines the uric acid-to-HDL cholesterol ratio (UHR), a novel inflammatory and metabolic marker, as a potential predictor of depression in women.

**Objective:**

To evaluate the association between UHR and depression risk in adult women.

**Methods:**

This pooled cross-sectional analysis included 7,925 women aged ≥20 years, using the combined 2005–2018 NHANES cycles. Depression was defined by a Patient Health Questionnaire-9 (PHQ-9) score ≥10. UHR was calculated as uric acid (mg/dL) divided by HDL cholesterol (mg/dL) multiplied by 100%. Multivariable logistic regression was adjusted for sociodemographic, clinical, and lifestyle confounders. Threshold effects and subgroup analyses were conducted to explore nonlinear relationships and robustness across population strata.

**Results:**

Elevated UHR showed a linear association with increased depression risk. Each unit increase in UHR corresponded to a 5% higher likelihood of depression (OR=1.05, 95% CI=1.02–1.09). Women in the highest UHR quartile had nearly double the depression risk compared to the lowest quartile (OR=1.97, 95% CI=1.40–2.77). A critical inflection point at UHR=8.12 indicated a 6% incremental risk per unit beyond this threshold. Subgroup analyses confirmed consistent associations across demographic and clinical groups, with heightened effects in women aged <45 years.

**Conclusion:**

Higher UHR levels are independently associated with depression in adult women, suggesting its utility as a metabolic-inflammatory biomarker for depression risk stratification. These findings highlight the interplay between lipid metabolism, oxidative stress, and mental health, advocating for UHR integration into preventive strategies for women’s mental well-being.

## 1 Introduction

Depression constitutes a major global public health concern, affecting approximately 280 million people and imposing significant burdens on individual well-being, familial relationships, and socioeconomic structures (1). This mood disorder, typified by persistent sadness and diminished participation in daily activities, demonstrates a clear gender disparity, with women exhibiting twice the risk compared to men. This increased susceptibility first emerges during adolescence and persists throughout adulthood (2). Biological factors such as genetic susceptibility, hormonal fluctuations, and neurohormonal sensitivity, along with psychosocial factors like role stress and gender-specific socialization, contribute to the heightened susceptibility of women to depression (3, 4).

Uric acid (UA) serves as a principal antioxidant within plasma, neutralizing over 50% of circulating free radicals. Its neuroprotective properties and role in stabilizing neuronal ascorbic acid underscore its relevance in neurological conditions, including depressive disorders (5). Estrogen facilitates UA excretion by making phospholipases in organelles more susceptible to urate crystals, countering crystallization (6). High-density lipoprotein cholesterol (HDL-C) occupies a pivotal role in cerebral cholesterol metabolism, facilitating reverse cholesterol transport essential for cholesterol clearance. Its anti-inflammatory and antioxidative capabilities offer neuroprotective effects and support functional regulation within the brain (7). Nevertheless, current literature reveals conflicting findings regarding HDL-C’s relationship with depression, ranging from positive to inverse or non-existent correlations, underscoring complex regulatory mechanisms requiring further investigation (8–10). In postmenopausal women, decreased estrogen levels may reduce HDL-C levels, leading to severe mood swings and cognitive impairment. HDL-C levels may partially reflect the clinical manifestations of depression (11). Growing evidence suggests the uric acid-HDL cholesterol ratio (UHR) as a holistic indicator of oxidative-inflammatory homeostasis (12). While demonstrating prognostic potential across metabolic disorders, its role in female affective pathology remains insufficiently characterized - a critical oversight demanding rigorous investigation to validate its discriminative capacity in mood disorder prediction.

By pooling eight consecutive NHANES cycles into one survey-weighted dataset, this study conducts a cross-sectional analysis to examine the association between UHR and depression in women. Through systematic evaluation of UHR-depression correlations in a nationally representative female cohort, this work provides mechanistic insights to guide early intervention protocols targeting neuro-metabolic pathways in depression etiology.

## 2 Methods

### 2.1 Study population and design

This research employed nationally representative data obtained from the NHANES database. The survey was thoroughly reviewed and received institutional approval. Ethical clearance for using the dataset was granted by the relevant institutional review boards, eliminating the necessity for further ethical consent.

**Figure 1:**
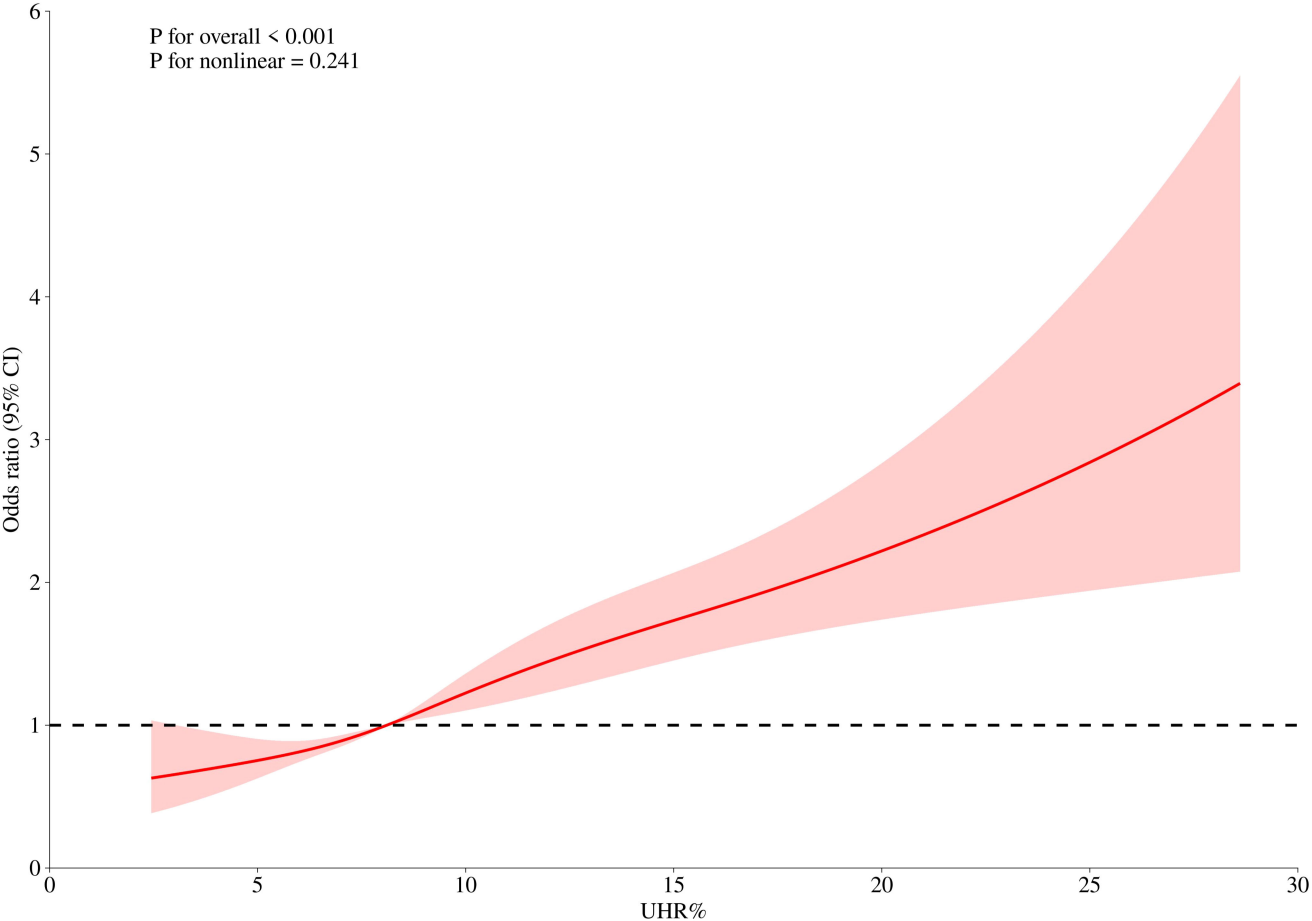
Diagram of participant enrollment process.

From the 2005–2018 datasets, we initially identified 70,190 subjects with relevant UHR and depression data. The inclusion criteria comprised women aged ≥20 years (n=31,105). Participants were excluded if data were incomplete for UA or HDL-C values (n=4,025), depression assessments (n=3,347), or relevant covariates (n=15,808), resulting in a final analytical cohort of 7,925 women. Ultimately, 7,925 eligible female participants were included (**Figure 1**).

### 2.2 Assessment of depression

Depression diagnoses were operationalized through PHQ-9 assessments aligned with DSM-IV nosology. The validated instrument employs a stratified scoring system (0–3 per item; cumulative range 0-27), with threshold scores ≥10 indicating clinically significant depressive symptomatology requiring intervention (13).

### 2.3 Evaluation of UHR

UHR was quantified from fasting blood samples collected during NHANES physical examinations (2005–2018). Biochemical quantifications followed CDC-standardized analytical methodologies. UHR computation employed the formula: UHR = [UA (mg/dL)/HDL-C (mg/dL)] × 100%.

### 2.4 Other covariates

Covariate selection encompassed three principal dimensions. First, questionnaire data provided information regarding participants’ age, race, educational background, marital status, alcohol consumption, family poverty-to-income ratio (PIR), and prevalent conditions that might influence UHR levels. These conditions encompassed hypertension, diabetes, stroke, high cholesterol, arthritis, congestive heart failure, emphysema, asthma, coronary artery disease, liver disorders, angina, thyroid abnormalities, myocardial infarction, and cancers. The participants were categorized into married and unmarried women based on their marital status. Alcohol consumption was assessed based on drinking frequency over the past 12 months and categorized as ‘no’ for participants who consumed fewer than 12 alcoholic beverages and ‘yes’ for those who consumed at least 12 beverages during this period. Household economic standing was quantified through the PIR. Laboratory and clinical measurements provided additional data, including smoking status, lipid concentrations, and body mass index (BMI). Smoking status was quantified via serum cotinine concentrations, a biomarker for tobacco exposure. We divided the serum cotinine levels into tertiles: low concentration, medium concentration, and high concentration, representing low, medium, and high levels of smoking status, respectively. Lipid levels were determined by measuring total cholesterol in the blood. BMI was calculated as kg/m² and classified into three categories: <25, 25-30, and >30. Dietary intake information was assessed through validated dietary recall instruments, which included total fat, protein, carbohydrate, and fiber intake, alongside specific vitamins such as B6, B12, C, and K. This comprehensive data collection allowed for a thorough assessment of dietary factors that might influence UHR levels and, consequently, depression risk in women.

### 2.5 Statistical analysis

All analyses incorporated NHANES’s multistage probability sampling framework, with appropriate statistical adjustments for oversampling, nonresponse patterns, and stratification effects. The NHANES dataset employs specialized weights to address the complex survey structure, including post-stratification adjustments based on census data. In this research, categorical variables were represented as weighted percentages, whereas continuous variables were presented as means along with their standard deviations. The differences in baseline characteristics were evaluated by applying weighted linear regression for continuous variables and weighted chi-square tests for categorical variables.

To investigate the association between UHR and depression, three multivariable logistic regression models were progressively constructed. Model 1 utilized a univariate approach, while Model 2 adjusted for key demographic confounders such as age, race, and education level. Model 3 further adjusted for alcohol consumption, PIR, marital status, BMI, cholesterol, aspartate aminotransferase, creatinine, triglycerides, UA, intake of various dietary fatty acids, and medical history variables (diabetes, hypertension, arthritis, high cholesterol, asthma, congestive heart failure, liver disease, coronary heart disease, thyroid disease, emphysema, myocardial infarction, malignancies, stroke). By comparing results across these three models we can assess the robustness of the association between UHR and depression as well as understand how adjustments for different covariates impact this relationship. Nonlinear relationships were explored via smoothed curve fitting, with threshold effects analyzed to identify critical UHR inflection points after covariate adjustment. Stratification analyses examined effect modification across population subsets, with multiplicative interaction terms testing subgroup heterogeneity. Analytical workflows were implemented in R 4.2.3 and EmpowerStats 2.0, applying sampling weights and design effects. Statistical significance was determined through bilateral hypothesis testing (α=0.05).

## 3 Results

### 3.1 Participant characteristics

After excluding individuals with incomplete data on UHR and depression, a final cohort of 7,925 women was included for analysis. Study participants were stratified into depressive and non-depressive cohorts using the PHQ-9 diagnostic threshold. The analysis revealed notable disparities in the distribution of covariates between these groups. Women with depression exhibited younger age at assessment, reduced poverty-income ratios, lower educational attainment, elevated body mass indices, higher prevalence of unpartnered status, and overrepresentation within the Non-Hispanic White demographic. Additionally, they exhibited higher serum triglyceride levels, elevated UHR values, and poorer lifestyle and dietary patterns (**Table 1**).

**Table 1:** Weighted comparison in basic characteristics.

Characteristics	Overall	Depression		P-value
		Without	With	
	n=7925	n=7136	n=789	
Age, years	47.23 ± 16.55	47.48 ± 16.76	44.96 ± 14.32	<0.001
PIR	2.82 ± 1.66	2.92 ± 1.64	1.92 ± 1.53	<0.001
Race(%)				<0.001
Mexican American	971 (12.25%)	884 (12.39%)	87 (11.03%)	
Other Hispanic	720 (9.09%)	616 (8.63%)	104 (13.18%)	
Non-Hispanic White	3878 (48.93%)	3531 (49.48%)	347 (43.98%)	
Non-Hispanic Black	1624 (20.49%)	1433 (20.08%)	191 (24.21%)	
Other Race	732 (9.24%)	672 (9.42%)	60 (7.60%)	
Education level(%)				<0.001
Less than 9th	314 (3.96%)	263 (3.69%)	51 (6.46%)	
9–11th	787 (9.93%)	656 (9.19%)	131 (16.60%)	
High school	1601 (20.20%)	1406 (19.70%)	195 (24.71%)	
Some college	2873 (36.25%)	2574 (36.07%)	299 (37.90%)	
College graduate	2350 (29.65%)	2237 (31.35%)	113 (14.32%)	
Marry(%)				<0.001
Unmarried	4113 (51.90%)	3565 (49.96%)	548 (69.46%)	
Married	3812 (48.10%)	3571 (50.04%)	241 (30.54%)	
Drinking(%)				<0.001
Low	7303 (92.15%)	6672 (93.50%)	631 (79.97%)	
High	622 (7.85%)	464 (6.50%)	158 (20.03%)	
Smoking(%)				<0.001
Low	2524 (31.85%)	2388 (33.46%)	136 (17.24%)	
Med	2758 (34.80%)	2544 (35.65%)	214 (27.12%)	
High	2643 (33.35%)	2204 (30.89%)	439 (55.64%)	
BMI(kg/m²)				<0.001
<25	2467 (31.13%)	2298 (32.20%)	169 (21.42%)	
25-30	2201 (27.77%)	2015 (28.24%)	186 (23.57%)	
≥30	3257 (41.10%)	2823 (39.56%)	434 (55.01%)	
Diabetes(%)				<0.001
Yes	681 (8.59%)	575 (8.06%)	106 (13.43%)	
No	7074 (89.26%)	6415 (89.90%)	659 (83.52%)	
Pre-diabetes	170 (2.15%)	146 (2.05%)	24 (3.04%)	
Hypertension(%)				<0.001
Yes	2608 (32.91%)	2274 (31.87%)	334 (42.33%)	
No	5317 (67.09%)	4862 (68.13%)	455 (57.67%)	
High Cholesterol(%)				0.005
Yes	2645 (33.38%)	2274 (31.87%)	334 (42.33%)	
No	5280 (66.62%)	4862 (68.13%)	455 (57.67%)	
Asthma(%)				<0.001
Yes	1374 (17.34%)	1176 (16.48%)	198 (25.10%)	
No	6551 (82.66%)	5960 (83.52%)	591 (74.90%)	
Arthritis(%)				<0.001
Yes	2334 (29.45%)	1994 (27.94%)	340 (43.09%)	
No	5591 (70.55%)	5142 (72.06%)	449 (56.91%)	
Congestive cardiac failure(%)				<0.001
Yes	133 (1.68%)	99 (1.39%)	34 (4.31%)	
No	7792 (98.32%)	7037 (98.61%)	755 (95.69%)	
Coronary heart disease(%)				0.001
Yes	144 (1.82%)	118 (1.65%)	26 (3.30%)	
No	7781 (98.18%)	7018 (98.35%)	763 (96.70%)	
Angina pectoris(%)				<0.001
Yes	157 (1.98%)	106 (1.49%)	43 (5.45%)	
No	7768 (98.02%)	7030 (98.51%)	746 (94.55%)	
Acute infarction(%)				0.002
Yes	157 (1.98%)	130 (1.82%)	27 (3.42%)	
No	7768 (98.02%)	7006 (98.18%)	762 (96.58%)	
Stroke(%)				<0.001
Yes	205 (2.59%)	156 (2.19%)	49 (6.21%)	
No	7720 (97.41%)	6980 (97.81%)	740 (93.79%)	
Pulmonary emphysema(%)				<0.001
Yes	95 (1.20%)	70 (0.98%)	25 (3.17%)	
No	7830 (98.80%)	7066 (99.02%)	764 (96.83%	
Liver disease(%)				<0.001
Yes	265 (3.34%)	204 (2.86%)	61 (7.73%)	
No	7660 (96.66%)	6932 (97.14%)	728 (92.27%)	
Malignant tumor(%)				0.004
Yes	1238 (15.62%)	1087 (15.23%)	151 (19.14%)	
No	6687 (84.38%)	6049 (84.77%)	638 (80.86%)	
Thyroid disease(%)				0.065
Yes	777 (9.80%)	685 (9.60%)	92 (11.66%)	
No	7148 (90.20%)	6451 (90.40%)	697 (88.34%)	
High-density lipoprotein(mg/dl)	59.41 ± 16.96	60.01 ± 16.99	54.01 ± 15.68	<0.001
Cholesterol(mg/dl)	197.93 ± 41.39	197.99 ± 41.19	197.37 ± 43.22	0.689
Aspartate transaminase(u/l)	23.33 ± 13.33	23.19 ± 12.46	24.60 ± 19.47	0.005
Creatinine(mg/dl)	0.77 ± 0.27	0.77 ± 0.25	0.79 ± 0.40	0.054
Triglyceride(mg/dl)	134.20 ± 92.22	131.92 ± 89.05	154.81 ± 115.02	<0.001
Uric acid(mg/dl)	4.82 ± 1.26	4.81 ± 1.25	4.95 ± 1.32	0.003
UHR(%)				<0.001
≤6.13	1979 (24.97%)	1851 (25.94%)	128 (16.22%)	
6.14-8.10	1972 (24.88%)	1790 (25.08%)	182 (23.07%)	
8.11-10.76	1983 (25.02%)	1799 (25.21%)	184 (23.32%)	
>10.76	1991 (25.12%)	1696 (23.77%)	295 (37.39%)	
Total fat intake(gm)	73.17 ± 37.97	73.49 ± 37.77	70.29 ± 39.70	0.025
Saturated fatty acid intake(gm)	23.50 ± 13.70	23.55 ± 13.65	23.02 ± 14.14	0.304
Monounsaturated fatty acid intake(gm)	25.82 ± 14.24	25.94 ± 14.16	24.74 ± 14.90	0.024
Polyunsaturated fatty acid intake(gm)	17.32 ± 11.01	17.45 ± 10.97	16.12 ± 11.33	0.001
Cholesterol intake(gm)	254.62 ± 198.07	256.68 ± 198.62	236.06 ± 192.21	0.006

### 3.2 Association between UHR and depression

Multivariable regression models demonstrated a dose-response relationship between UHR elevation and depressive risk (**Table 2**). In the crude model, each UHR unit increment corresponded to an 8% increased likelihood of depression (OR=1.08, 95%CI 1.06–1.09). Subsequent adjustment for demographic confounders (Model 2: OR=1.07, 95%CI 1.05–1.09) and comprehensive covariates including health behaviors (Model 3: OR=1.05, 95%CI 1.02–1.09) preserved statistical significance (all *P*<0.01), confirming UHR’s independent predictive capacity.

**Table 2:** Association between UHR and depression in women.

	Continuous	Q1	Q2	Q3	Q4
Model 1 OR (95% CI)	1.08 (1.06, 1.09)	1	1.47 (1.16, 1.86)	1.48 (1.17, 1.87)	2.52 (2.02, 3.13)
Model 2 OR (95% CI)	1.07 (1.05, 1.09)	1	1.40 (1.10, 1.77)	1.34 (1.06, 1.70)	2.25 (1.80, 2.81)
Model 3 OR (95% CI)	1.05 (1.02, 1.09)	1	1.41 (1.09, 1.83)	1.26 (0.95, 1.68)	1.97 (1.40, 2.77)

Quartile-based analysis revealed substantial risk stratification, with the uppermost UHR quartile demonstrating markedly elevated odds compared to the reference quartile (OR = 1.97; 95% CI, 1.40–2.77). To further explore this relationship, we conducted smooth curve fitting and threshold effect analyses.

### 3.3 Smooth curve fitting and threshold effect between UHR and depression

**Figure 2:**
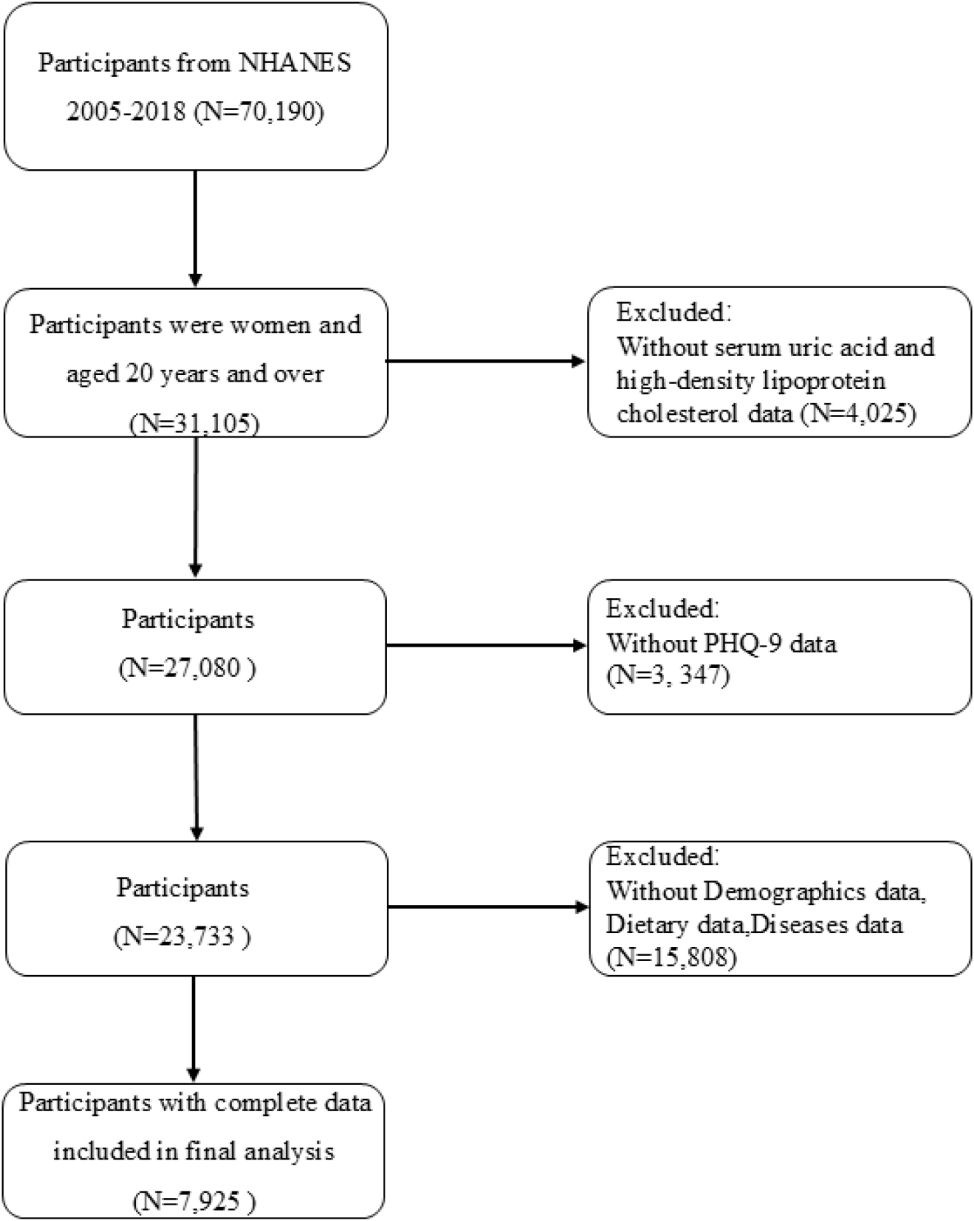
The association between UHR and depression in women.

To further clarify the relationship between UHR and depression, we used restricted cubic spline (RCS) curve analysis and threshold effect evaluation (**Table 3**, **Figure 2**). The findings showed a linear relationship between UHR and depression, with no indication of a threshold effect. However, for UHR values exceeding 8.12, each additional unit increase was associated with a 6% higher risk of depression (OR = 1.06; 95% CI, 1.03–1.08), suggesting that elevated UHR levels may contribute to the onset of depression.

**Table 3:** Analysis of threshold effect.

UHR	Adjusted OR (95% CI), *P* value
Model 1	
A straight-line effect	1.06 (1.04, 1.08) <0.01
Model 2	
Fold points (K)	8.12
UHR ≤ 8.12	1.07 (1.00, 1.14) 0.06
UHR> 8.12	1.06 (1.03, 1.08) <0.01
Effect size difference of 2 versus 1	0.99 (0.92, 1.07) 0.83
Equation predicted values at break points	-2.50 (-2.59, -2.40)
Log likelihood ratio tests	0.83

### 3.4 Subgroup analysis

**Figure 3:**
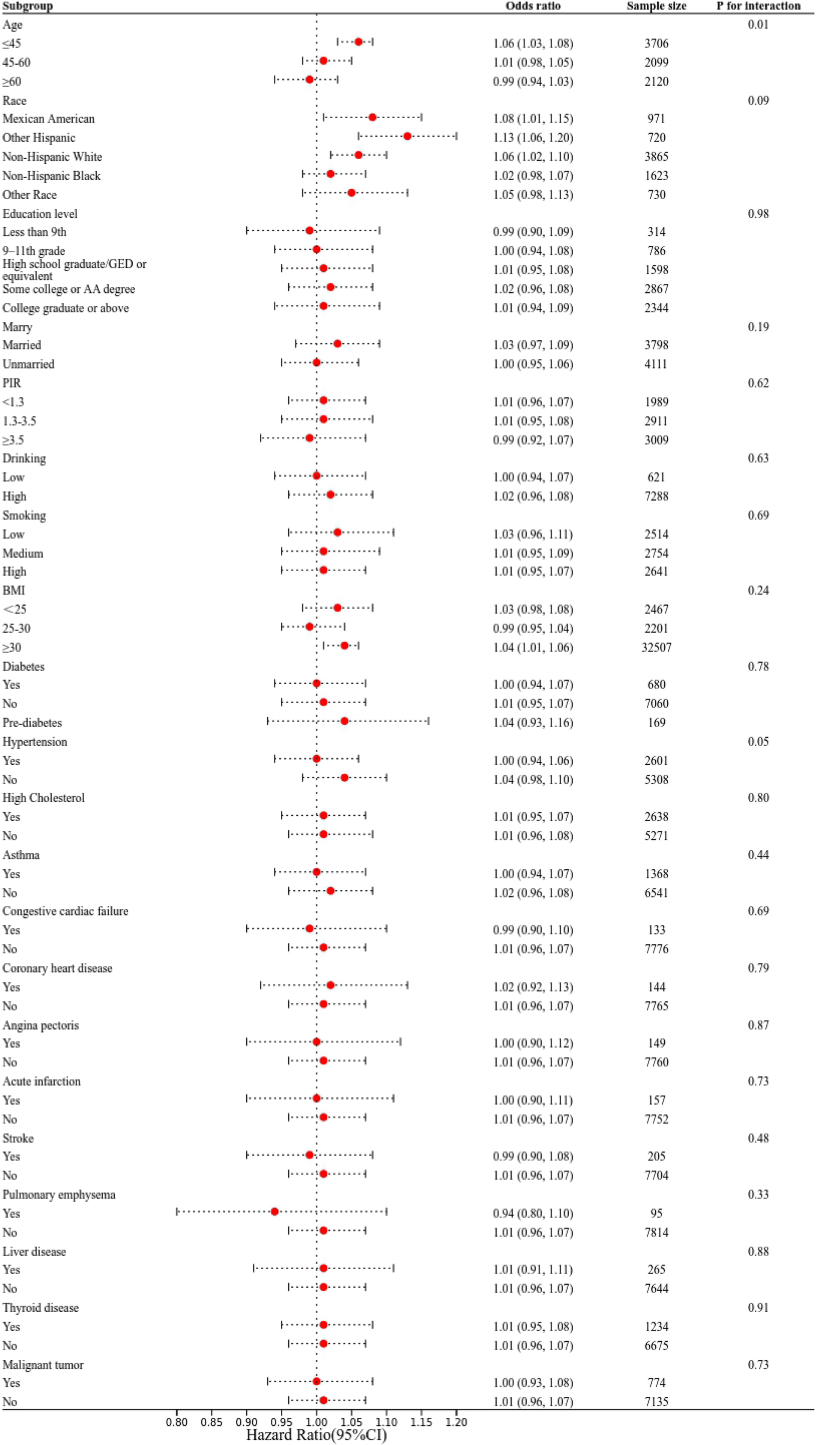
Subgroup analysis for the association between UHR and depression in women.

Subgroup analyses were performed to evaluate the consistency of the relationship between UHR and depression across different demographic and clinical groups (**Figure 3**). Interaction tests revealed no significant variation in the association between UHR and depression across categories defined by race, education, marital status, smoking, alcohol consumption, PIR, BMI, and various health conditions (all interaction *P* > 0.05). Notably, age-stratified analyses revealed persistent dose-response relationships between UHR and depression risk across all examined age stratifications (<45/≥45, <60/≥60), with non-linear threshold effects maintaining statistical significance through multiple analytical approaches.

## 4 Discussion

This nationwide representative survey involving 7,925 female participants revealed a novel epidemiological association between the UHR and clinically significant depressive symptoms. Our results show a strong linear correlation between UHR and depression, consistent across analyses treating UHR as both continuous and categorical variables. This suggests that interventions to reduce UHR could be beneficial at any level, not just for those with extremely high values. Our smooth curve fitting and threshold effect analyses identified a critical UHR threshold of 8.12, beyond which each unit increase in UHR corresponds to a 6% increase in depression risk. This finding indicates a potential clinical threshold that warrants increased vigilance from healthcare providers. Professionals may consider more intensive monitoring or earlier interventions for patients whose UHR levels approach or exceed 8.12. Notably, this association persisted even after adjusting for various potential confounders, underscoring the robustness of the relationship. The mechanistic implications of UHR-depression pathophysiology present compelling opportunities for preventive medicine strategies. Targeted interventions modulating purine metabolism and lipoprotein profiles - whether through nutraceutical approaches or emerging metabolic therapies - could potentially modify depression trajectories, offering dual cardiometabolic and neuropsychiatric benefits.

Overall, compared to individual markers such as UA or HDL-C, UHR may capture the interaction between UA and HDL-C more effectively, thereby reflecting inflammatory metabolic characteristics. As an integrated biomarker reflecting lipid-peroxidation interplay, UHR demonstrates significant clinical validity for depression risk stratification in female populations. A case-control investigation comparing biochemical profiles of 104 female major depressive disorder (MDD) patients with 52 healthy counterparts revealed significant associations between hyperuricemia and suicidal ideation severity in MDD women. The findings demonstrated a significant association between reduced serum uric acid levels and an elevated risk of suicide in patients with MDD, suggesting that dysfunction within the purinergic system may underlie the heightened suicide risk observed in female patients (14). A cross-sectional analysis of 32,424 U.S. adults from NHANES 2005–2018 revealed a nonlinear inverse trend: compared to the lowest UA quartile, the 2nd, 3rd, and 4th quartiles exhibited 9 %, 14.6 %, and 20.5 % lower odds of depressive symptoms, respectively (15). In a Japanese community sample (n = 705), lower UA levels were significantly associated with depression (OR = 0.816, 95 % CI, 0.673-0.988) (16). These converging findings underscore UA’s protective or inverse relationship with depression via antioxidant mechanisms. A Korean analysis investigating the associations between biochemical and anthropometric markers and depressive disorders in males and females included 33,993 participants. Results showed that in women, the depression group had higher levels of triglycerides, AST, blood urea nitrogen, and creatinine, and lower levels of HDL-C, hematocrit, and red blood cell counts (17). Peripartum lipid dynamics emerge as critical modulators of postpartum affective states. An Indian cohort study demonstrated inverse associations between HDL-C concentrations and postpartum depression (PPD) severity, with hypocholesterolemia predicting poorer psychometric outcomes on standardized assessments. This further validates the role of serum lipid levels in PPD among women, potentially providing biological targets for future new therapeutic strategies (18). Moreover, a recent study found that low HDL-C levels were significantly associated with increased depressive symptoms and cognitive impairment. These associations were consistently observed in both a multigenerational cohort and NHANES participants, underscoring HDL-C’s independent role in mood and cognitive dysregulation (19). Four large-scale studies published in 2025 have further elucidated the metabolic–psychiatric relevance of the UHR. Two NHANES-based analyses reported a positive association between UHR and depression risk among U.S. adults, with 36% and 52% higher odds observed in the fourth quartile and fifth quintile, respectively, compared to the lowest quartile (20, 21). This dose–response trend supports UHR as a potential biomarker for identifying individuals at elevated risk of depression. In contrast, a CHARLS-based study of 10,396 Chinese participants found an inverse association: each unit increase in UHR was linked to a 14% reduction in depressive symptoms, most notably among women aged ≥60 years (22). Beyond psychiatric outcomes, a 20-year cohort study involving 29,742 adults aged ≥40 years demonstrated that elevated UHR predicted both all-cause mortality (HR = 1.02) and cardiovascular mortality (HR = 1.03), highlighting its systemic prognostic value (23). Because UA can act as a pro-oxidant inside cells while HDL-C exerts anti-oxidant, anti-inflammatory effects, the UHR integrates these opposing forces and serves as a composite indicator of redox imbalance. Our findings, along with recent NHANES and CHARLS data on UHR, support its role as a dual-pathway biomarker that integrates purinergic and lipid-related mechanisms. This reinforces the relevance of UHR at the metabolic–psychiatric interface, particularly in women experiencing hormonal shifts that influence both UA and HDL-C.

In females, a strong association exists between metabolic dysregulation and depressive disorders (3, 24–26). Sex hormones in women, especially estrogen, significantly affect lipid metabolism, and lipid metabolism abnormalities may reflect a chronic inflammatory state in the body (27, 28). The interaction between estrogen and inflammatory responses may influence inflammatory reactions in the brain and neuronal health by regulating the metabolism of fatty acids, particularly polyunsaturated fatty acids (29, 30). Consequently, fluctuations in estrogen levels, particularly during specific physiological states such as menopause and pregnancy, may lead to inflammatory responses that potentially increase the risk of depression in women. Our subgroup analysis revealed significant interactions between UHR and depression across age groups (<45/≥45, <60/≥60 years), further supporting the robustness of this relationship. This suggests that the impact of UHR levels on depression may be more pronounced in individuals of different age groups. In women of childbearing age, estrogen and progesterone are the main sex hormones and play crucial roles in regulating metabolism, lipid profiles, immune responses, and more (31–33). Estrogen not only promotes serotonin synthesis but also enhances serotonin receptor sensitivity. Fluctuations in estrogen levels may inhibit serotonin synthesis and receptor function, leading to mood instability and increased risk of depression (34, 35). Endocrinological transitions across the female lifespan - particularly progesterone-mediated alterations in adipocyte biology and insulin signaling - may fundamentally modify lipid-uric acid homeostasis (36, 37). These age-related endocrine variations necessitate stratified clinical interpretation of UHR’s depression risk predictions. When employing UHR as a biomarker to evaluate its association with depression in women, this factor should be taken into consideration to elucidate the complex relationships and potential biological mechanisms underlying the interplay between age, UHR, and depressive disorders in the female population. Interestingly, although obesity is linked to mood disorders, our stratified analyses showed no significant interaction between BMI and UHR, indicating a stable association across BMI groups. Further studies are needed to explore BMI’s moderating role.

Moreover, prior research has predominantly concentrated on examining the relationships between physical activity, environmental exposures, lifestyle factors, and their impact on depression. Contemporary neuro-epidemiological research has elucidated modifiable risk pathways connecting lifestyle behaviors, environmental exposures, and affective disorder pathogenesis through metabolic-inflammatory mechanisms. However, the focus has largely been on individual variables, often overlooking the complex interplay between metabolic markers, such as UHR, and mental health outcomes (38–40). Given that UHR is routinely available from standard biochemical panels, its incorporation into regular health assessments presents a cost-effective tool for early depression risk screening, especially in individuals nearing the identified risk threshold of 8.12%. Lifestyle modifications that concurrently lower uric acid and raise HDL-C may offer accessible and biologically plausible interventions to reduce depressive symptoms. Moreover, age- and ethnicity-specific variations, as observed in recent studies, call for tailored UHR reference ranges in diverse populations (22, 23). Collectively, these insights emphasize the clinical value of UHR in bridging metabolic and psychiatric care, and underscore the need for prospective trials to assess whether pharmacological or lifestyle-based modulation of UHR can yield tangible benefits for both mental health. Our research evaluated a comprehensive range of indicators within UHR that are closely associated with lipid metabolism. This broader approach allowed us to explore potential links between lipid metabolism and depression. The demonstrated metabolic-psychiatric synergies underscore the clinical imperative for optimizing metabolic homeostasis in women’s mental healthcare paradigms, particularly through proactive management strategies targeting subclinical inflammation and oxidative stress pathways.

## 5 Limitations and strengths

Despite its strengths, this study has several limitations that warrant consideration. First, while the PHQ-9 demonstrates robust psychometric properties for depressive-symptom screening, its reliance on subjective self-reports introduces potential measurement bias. Second, because our analytic sample was restricted to individuals aged ≥ 20 years, the extrapolation of findings to adolescent or pediatric populations should be approached with caution. Third, we did not perform single-marker analyses modeling serum UA and HDL-C separately, which warrants future research to disentangle their individual contributions to depression risk. Although subgroup analyses and interaction tests help reveal differences among groups, these results require further validation through additional studies. Particularly regarding its relevance across various physiological and pathological conditions, to improve the robustness of the results. Our analysis adopts a population-based cross-sectional design to systematically examine correlations between the UHR and depressive morbidity within a nationally representative female cohort. Prospective cohort studies would better establish the temporal relationships and enhance the predictive capacity of UHR for forecasting depression in women. Finally, to decipher the pathophysiological interplay, subsequent investigations should integrate translational approaches combining preclinical models with human cohort data. Such methodology could elucidate the tripartite dynamics of estrogen signaling, UHR modulation, and neuroendocrine pathways in depression etiology. Animal models can offer valuable insights into the molecular and physiological pathways involved, which can complement and guide human clinical studies. Such studies would enable a thorough investigation into the mechanisms and foundational principles governing their interaction. Multidimensional investigations of this nature could elucidate the pathophysiological interdependencies between metabolic and inflammatory markers and depression, potentially leading to novel and more effective strategies for both prevention and treatment of depression and its associated complications.

Despite certain limitations, the study boasts several methodological merits. UHR, as an accessible and straightforward biomarker, serves as a valuable tool for exploring its link with depression. To mitigate confounding factors and strengthen internal validity, we selected demographically matched cohorts for analysis. Female-specific sampling minimizes the influence of gender disparities, allowing for a more accurate examination of the relationship between UHR and depression. Moreover, the use of NHANES data, drawn from a stratified probability sampling framework, ensures population-level generalizability and robust external validity, particularly within the non-institutionalized U.S. demographic. Multivariable adjustment for demographic, metabolic, and behavioral confounders strengthens the specificity of observed UHR-depression associations. The epidemiological relevance of these associations underscores UHR’s potential utility in population-level mental health risk stratification frameworks.

## 6 Conclusion

This study identifies UHR as an integrative biomarker reflecting concurrent dyslipidaemia and subclinical inflammation in female depression pathogenesis. The complexity of UHR’s biological interactions demands systematic investigation through international collaborative cohorts employing prospective designs. Such efforts will clarify temporal relationships and strengthen causal inference regarding UHR’s role in depressive disorder development. Clinically, considering incorporating UHR levels into depression risk assessment systems may help in the early identification of high-risk female patients, enabling earlier and more personalized interventions. Future precise biomarker monitoring is expected to optimize prevention and treatment strategies for depression in women.

## Data Availability

The original contributions presented in the study are included in the article/Supplementary Material. Further inquiries can be directed to the corresponding author.
